# Value of MRI signal intensity in evaluation of allergic fungal rhinosinusitis compared with CT Hounsfield units: Retrospective study

**DOI:** 10.1097/MD.0000000000038951

**Published:** 2024-07-12

**Authors:** Seham Alsalem, Ali Almontashri, Mohammed Alsalem, Fahad Altamimi, Nasher Alyami, Shaker Hajjaf, Faisal Ahmed

**Affiliations:** a Department of Radiology, Prince Sultan Military Medical City, Riyadh, Saudi Arabia; b Department of Radiology, King Saud Medical City, Riyadh, Saudi Arabia; c Department of Hematology, Maternity and Children Hospital, Najran, Saudi Arabia; d Department of Otolaryngology, King Saud Medical City, Alfaisal University, Riyadh, Saudi Arabia; e Department of Laboratory Medicine, Hematology Section, King Khaled University Hospital, Ministry of Health, Najran, Saudi Arabia; f Department of Radiology, King Saud Medical City, Ministry of Health, Riyadh, Saudi Arabia; g Department of Urology, School of Medicine, Ibb University, Ibb, Yemen.

**Keywords:** allergic fungal rhinosinusitis, computed tomography, diagnosis, Hounsfield units, magnetic resonance imaging, signal intensity, T2 mapping

## Abstract

“Allergic fungal sinusitis (AFS)” is typically diagnosed using radiologic images like computed tomography (CT) scans and magnetic resonance imaging (MRI), with the “Hounsfield unit (HU)” in CT scans and T2-weighted images (T2WI) in MRI serving as reliable objective parameters. However, diagnosing AFS might be difficult because of possible signal changes and densities caused by variations in the secretion concentration in the sinus. Few studies have compared the diagnostic performance of MRI and CT scans. This study aimed to investigate the value of MRI signal intensity in evaluating AFS compared with CT HUs. This retrospective study included 111 patients with pathologically confirmed AFS who underwent CT imaging followed by MRI evaluation at King Saud Medical City, Riyadh, Saudi Arabia, from January 2012 to December 2022. Radiographic densities of sinus opacities on CT scan, including the mean HU values, and MRI findings, including signal voids on T1-weighted images and T2WI, were gathered and analyzed. To determine the efficacy of these radiographic characteristics in predicting the disease and the best cutoff value, we employed receiver operator characteristic curves. The mean age was 31.9 ± 15.6 years, and most patients were 74 females (66.7%). The main symptom was nasal obstruction in 73 patients (65.8%). In comparison, between HU and signal void on T2WI, there was moderate predictive performance [area under the curve: 0.856, *P* = .001]. An ideal HU cutoff value of 69.50 HU was obtained with a sensitivity of 100% and a specificity of 44.7%. However, the receiver operator characteristic for T1-weighted images could not be plotted, as no signal was avoided to predict AFS and it was not statistically significant (area under the curve: 0.566; *P* = .287). The study found a CT HU of 69.5 can predict MRI T2WI signal values with a void signal, aiding in diagnostic workup and evaluation for AFS.

## 1. Introduction

Fungal sinusitis refers to a wide spectrum of fungal diseases that affect approximately 20% of the population, with a wide range of clinical manifestations ranging from mild irritation to serious health problems and death.^[1,2]^ There are several types of fungal sinusitis with classifications based on the degree of sinus invasion. There are 2 broad classifications of fungal sinusitis: noninvasive and invasive. The former has 3 unique subtypes: fungal balls (FB), saprophytic fungal sinusitis (SFS), and “allergic fungal sinusitis (AFS).”^[3]^ AFS accounts for 5 to 10% of all chronic rhinosinusitis cases. Patients frequently report chronic rhinosinusitis, nasal polyps, inhaled atopy, and high total serum immunoglobulin E (IgE). Affected sinuses are typically clogged with inspissated brown or greenish-black allergic mucus. The extra mucosal “peanut buttery” allergic mucin contains intact and degenerating eosinophils, Charcot-Leyden crystals, cellular debris, and scant fungal hyphae.^[4]^

Over time, AFS has become an underdiagnosed clinical condition. An accurate diagnosis is required for appropriate therapy, which is often determined by a combination of characteristic clinical and radiologic imaging, notably computed tomography (CT) and magnetic resonance imaging (MRI), histopathological findings, and immunologic characteristics of the disease.^[4,5]^ Previous studies have shown CT findings typical of AFS, including sinus opacification, mucocele formation, skull base erosion, and a hyper-attenuating AFS in the central sinus area.^[6–8]^ Other studies have assessed the accuracy of objective measurements of sinus opacity densities, including Hounsfield units (HU), in predicting noninvasive chronic rhinosinusitis.^[9–11]^ The reported MRI findings of AFS included low signal intensity on T1-weighted images (T1WI) and a signal void on T2-weighted imaging (T2WI) MRI (due to dense fungal concretions and heavy metals).^[6,12,13]^ Because of its widespread availability, quick scan duration, and capacity to examine many organs simultaneously, CT scans are the most often utilized imaging modality for initial AFS diagnosis. However, few investigations have focused on the MRI findings of the AFS.^[14]^ The “Hounsfield unit (HU)” in CT scans is a standardized objective unit that may be used to measure remodeling and opacification in CT scans of bone sections in patients with AFS.^[10]^ Furthermore, the signal loss in the core of the sinuses on T2WI in MRI corresponds to the central hyperdensity (high-attenuation areas) shown on CT scans, which is a feature of AFS.^[15]^ However, identifying AFS may be difficult because of changes in the signals and densities produced by differences in secretion concentration in the sinus. As a result, we anticipated that the AFS signal intensity MRI value would correspond with the HU in the CT scan and may predict the diagnosis. This study investigated the prediction accuracy of objective measurements of radiographic density (HU) of paranasal sinus opacities obtained from preoperative sinus CT images to identify and forecast MRI signal intensities for diagnostic purposes.

## 2. Material and methods

### 2.1. Study design and exclusion criteria

A retrospective study included 111 patients with pathologically confirmed AFS who underwent preoperative CT followed by MRI evaluation at King Saud Medical City, Riyadh (KSMC), located in Riyadh, Saudi Arabia, from January 2012 to December 2022. Patients with incomplete data and those with no CT or MRI records were excluded.

### 2.2. Computed tomography (CT) scan image and protocol

Sinus CT images were evaluated using the Sante DICOM and Communications in Medicine viewer, and sinus soft tissue densities were detected using the elliptical selection tool.^[9]^ Within each sinus opacity, the largest and most representative region of interest was measured, eliminating pockets of air, “frothy” patches, and bone or teeth. A minimum opacity size of 100 pixels was specified.^[9,16]^ The average CT HU (HU avg) is a density measure in Hus.^[9,17]^

### 2.3. Magnetic resonance imaging (MRI) protocol and imaging analysis

Magnetic resonance imaging was performed using a 1.5-T unit (Signa Twin Speed Excite, GE Healthcare, Chicago, IL) with an 8-channel head coil. After an intravenous injection of 0.1 mmol/kg of gadopentetate dimeglumine (Magnevist, Schering, Berlin, Germany), contrast-enhanced axial, coronal, and sagittal T1-weighted spin-echo images were obtained. In the axial plane, contrast-enhanced T1-weighted imaging was used, with frequency-selective fat saturation. The imaging parameters of PNS MR were repetition time (TR)/echo time (TE), 3000/126 ms; field of view (FOV), 194 × 230 mm; matrix, 512 × 281; and slice thickness/gap, 4/1.2 mm for T2WI and TR/TE, 598/11 ms; FOV, 194 × 230 mm; matrix, 384 × 211; and slice thickness/gap, 4/1.2 mm for T1WI. Routine brain MR parameters on 3T (MAGNETOM Skyra; Siemens Healthcare) were TR/TE, 5190/91 ms; FOV, 194 × 230 mm; matrix, 448 × 265; and slice thickness/gap, 5/2 mm for T2WI and TR/TE, 350/3 ms; FOV, 195 × 230 mm; matrix, 480 × 228; and slice thickness/gap, 5/2 mm for T1WI. The MRI protocol and imaging analysis were performed as previously reported by Wang et al^[18]^

### 2.4. Data collection

The collected data included demographic data (age and sex), main symptoms at presentation, Hounsfield unit on CT scan, presence or absence of low signal intensity on magnetic resonance imaging T1-weighted images (MRI T1WI), and presence or absence of signal void on MRI T2WI.

### 2.5. Image analysis

The images were examined separately by 2 competent radiologists using the consensus approach. Reviewers determined (1) the soft tissue mass’s location, (2) multifocality, and (3) the HU on CT scans. In terms of MR imaging, T1WI and T2WI sequences were examined. Then, we assessed the signal strength of AFS on T1WI and T2WI as follows: signal void, hyposignal intensity, hyper signal intensity, and heterogeneous signal intensity. Then, we compared the HU obtained by CT scan with observed the existence of the signal intensity component in T2WI and T1WI MRI.

### 2.6. Main outcome

To determine the efficacy of T1WI and T2WI signal intensity MRI images in comparison with HU values obtained by CT scans with reference to histopathological findings.

### 2.7. Statistical analysis

For quantitative data, mean and standard deviation were utilized, whereas frequency and percentage were employed for qualitative variables. Weighted κ statistics were used to evaluate inter-observer agreement in evaluating HU on CT scans and MRI signal intensities based on signal voids on T2WI and T1WI images. ROC curves were interpreted for sensitivity, specificity, and accuracy. The area under the receiver operator characteristic (ROC) curve (AUC) was used to assess diagnostic performance, with AUC values >0.9, 0.7 to 0.9, and 0.5 to 0.7 indicating strong, moderate, and low predictive capacity, respectively. In addition, the parameter value at the maximum Youden index was used as the threshold for judging AFS, and the corresponding sensitivity, specificity, and accuracy were calculated. Statistical significance was set at 0.05. IBM SPSS version 25 software (IBM Corp., Armonk, NY) was used for statistical analyses.

### 2.8. Ethical approval

Ethical approval was obtained from the ethics committee of King Saud Medical City (IRB Registration Number with KACST, KSA: H-01-*R*-053 and IRB Registration Number U.S. Department of HHS IORG #: IORG0010374; June 14, 2023) in adherence to the Declaration of Helsinki. Informed consent was obtained from all participants, emphasizing their voluntary participation, anonymity, and confidentiality.

## 3. Result

### 3.1. Characteristics of study subjects

**Figure 1.**
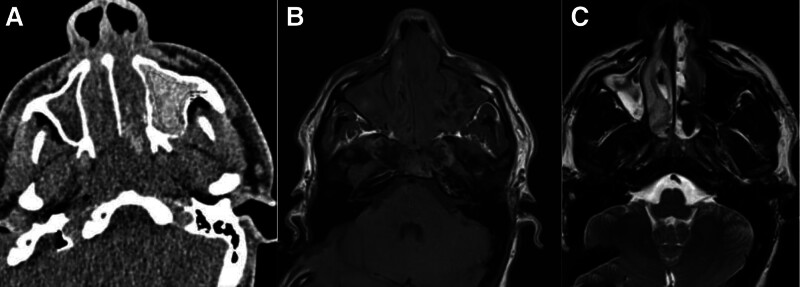
A 22-year-old male presented with a headache and signs of nasal obstruction. (A) An axial CT scan showed complete opacification of the paranasal sinuses, as seen in the bilateral maxillary sinuses and nasal cavity, with a high density of 119 HU in the left maxillary sinus. (B) Non-contrast MRI T1WI showed iso-signal intensity with foci of low signal intensity in the left maxillary sinus and (C) signal void T2WI with mixed density in the right maxillary sinus. Histopathology-proven fungal sinusitis. CT = computed tomography scan, HU = Hounsfield unit, MRI T1WI = magnetic resonance imaging T1-weighted images.

**Figure 2.**
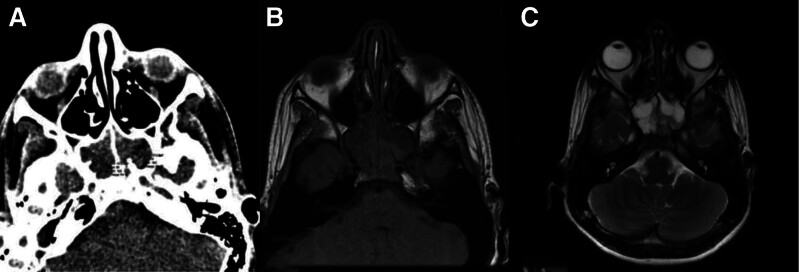
A 15-year-old male presented with a severe headache. (A) An axial CT scan showed expansion and complete opacification of bilateral sphenoid sinuses with a density of 29 and 31 HU in the right and left sphenoid sinuses, respectively. (B) Non-contrast MRIT1WI showed corresponding iso-signal intensity opacities with (C) predominantly high signal intensity in MRI T2WI. Histopathology has proven nonfungal. CT = computed tomography scan, HU = Hounsfield unit, MRI T1WI = magnetic resonance imaging T1-weighted images.

**Figure 3.**
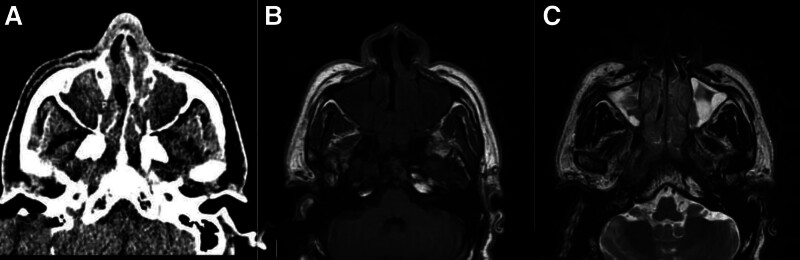
A 43-year-old male presented with a loss of smell and signs of nasal obstruction. (A) An axial CT scan showed complete opacification of bilateral maxillary sinuses with a density of 36 HU. (B) Non-contrast MRI T1WI showed isos-signal intensity corresponding to maxillary sinus opacities and (C) MRI T2WI showed areas of low and high signal intensities. Histopathology has proven a carcinoid tumor. CT = computed tomography scan, HU = Hounsfield unit, MRI T1WI = magnetic resonance imaging T1-weighted images.

A total of 111 patients met our inclusion criteria and were included in this study. The mean age was 31.9 ± 15.6 years (range 18–72 years), and most patients were female (n = 75, 66.7%). The main symptoms were nasal obstruction in 73 (65.8%) cases, followed by fungal sinusitis in 50 (45.0%) cases (Table 1). The mean HUs on the CT scan were 248.4 ± 124.4 HU (range 43.0 to 435.0 HU) (Figs. 1–3). Inter-observer agreement for signal void evaluation on T2WI and T1WI was significant and nearly perfect (κ = 0.78 and 0.89, respectively).

**Table 1:** Patients’ demographic characteristics of patients.

Variables	N (%)
Age, mean ± SD	31.9 ± 15.6 (range 18–72)
*Gender*	
Female	74 (66.7%)
Male	37 (33.3%)
*Sinus involvements*	
*Solitary involvements*	74 (66.7%)
Maxillary	52 (46.8%)
Sphenoidal sinus	22 (19.8%)
*Multiple involvements*	37 (33.3%)
Maxillary + ethnoidal sinuses	10 (9.0%)
Ethmoidal + sphenoidal sinuses	14 (12.6%)
Bilateral maxillary sinuses	9 (8.1%)
Bilateral sphenoidal sinuses	4 (3.6%)
*Main symptoms* *	
Headache	77 (69.4%)
Rhinorrhea	55 (49.5%)
Nasal dripping	51 (45.9%)
Nasal obstruction	41 (36.9%)
Hyposmia	16 (14.4%)
*Comorbidities*	
History of malignancy	5 (4.5%)
Diabetes mellitus	4 (3.6%)
Immunosuppression	3 (2.7%)
Hounsfield units, mean ± SD	248.4 ± 124.4 (range 43.0–435.0)

* Some patients had multiple symptoms.

### 3.2. Association between signal void on MRI T2WI and HU in CT scan

**Figure 4.**
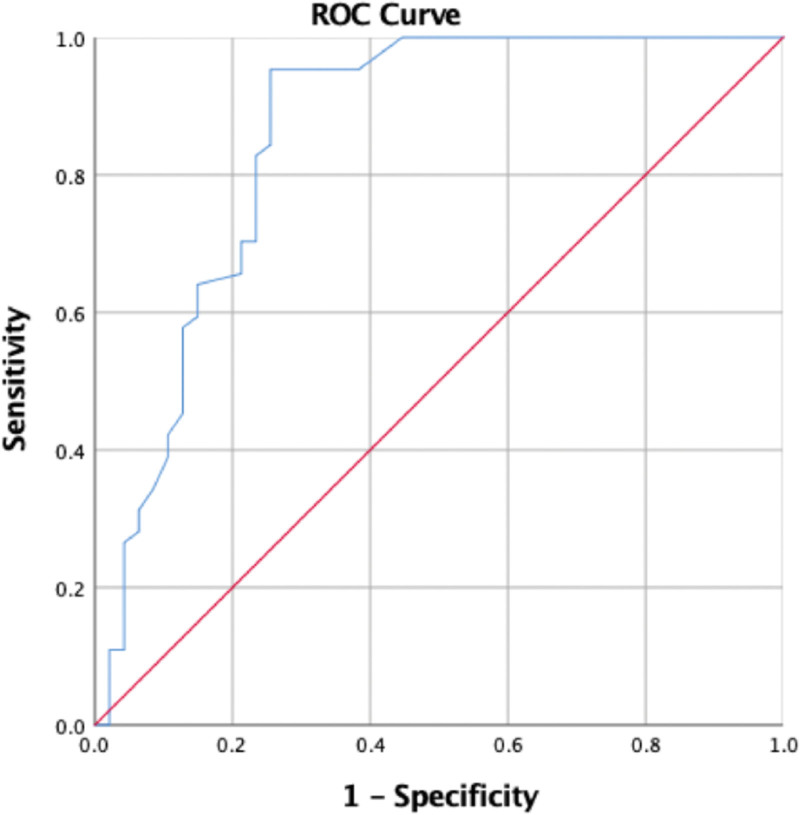
When the ROC curves of CT HU and MRI T2WI signal void are compared (where signal void is coded as 1, whereas all other values are coded as 0), the AUC is high around 0.856, and shows a significant *P*-value (*P* = .001). An ideal CT HU cutoff value of 69.50 is obtained with a sensitivity of 100% and specificity of 44.7%. CT = computed tomography scan, HU = Hounsfield unit, MRI T1WI = magnetic resonance imaging T1-weighted images, ROC = receiver operator characteristic.

The association between HU obtained by CT and signal intensity on T2WI was statistically significant (*P* < .001) (Table 2). When HU in the CT scan and signal void on MRI T2WI were compared (where the signal void was coded as 1, whereas all other values were coded as 0), the T2WI value was positively correlated with HU (*R* = 0.43, *P* < .001). Furthermore, ROC curve analysis showed moderate predictive performance. HU yielded a significantly higher AUC value of 0.856 (95% confidence interval, 0.725 to 0.901, *P* = .001) in the ROC curve analysis. This also resulted in higher sensitivity (95.31%) and specificity (74.47%). The Youden index was used to select the best cutoff point based on the optimal sensitivity and specificity to discriminate AFS, showing that the threshold of HU value distinguishing AFS was 69.50 HU, with a sensitivity of 100%, specificity of 44.7%, and accuracy of 77.6% (Fig. 4). This shows that with the signal void in MRI T2WI, HU in the CT scan can be used to predict AFS.

**Table 2:** Correlation between T2-weighted image and Hounsfield unit.

T2-weighted images	Total	Hyposignal intensity (N = 16)	Signal void (N = 64)	Heterogeneous signal intensity (N = 20)	Hypersignal intensity (N = 11)	*P*-value
Hounsfield unit, Mean (SD)	89.7 (31.3)	75.6 (37.6)	105.8 (17.2)	72.0 (33.1)	49.1 (17.4)	<.001

### 3.3. Association between the low signal intensity on MRIT1WI and HU

The association between HU obtained by CT and signal intensity on T1WI was not statistically significant (*P* = .859) (Table 3). In the assessment of AFS, the ROC curve cannot be plotted for “signal void,” as no MRI T1WI shows this signal intensity. Furthermore, the T1WI value was negatively correlated with HU (r = −0.35, *P* = .278).

**Table 3:** Correlation between T1-weighted image and Hounsfield unit.

T1-weighted images	Total	Hyposignal intensity (N = 60)	Hypersignal intensity (N = 30)	Heterogeneous signal intensity (N = 21)	*P*-value
Hounsfield unit, mean (SD)	89.7 (31.3)	92.1 (30.5)	88.3 (31.3)	90.5 (33.8)	.859

**Figure 5.**
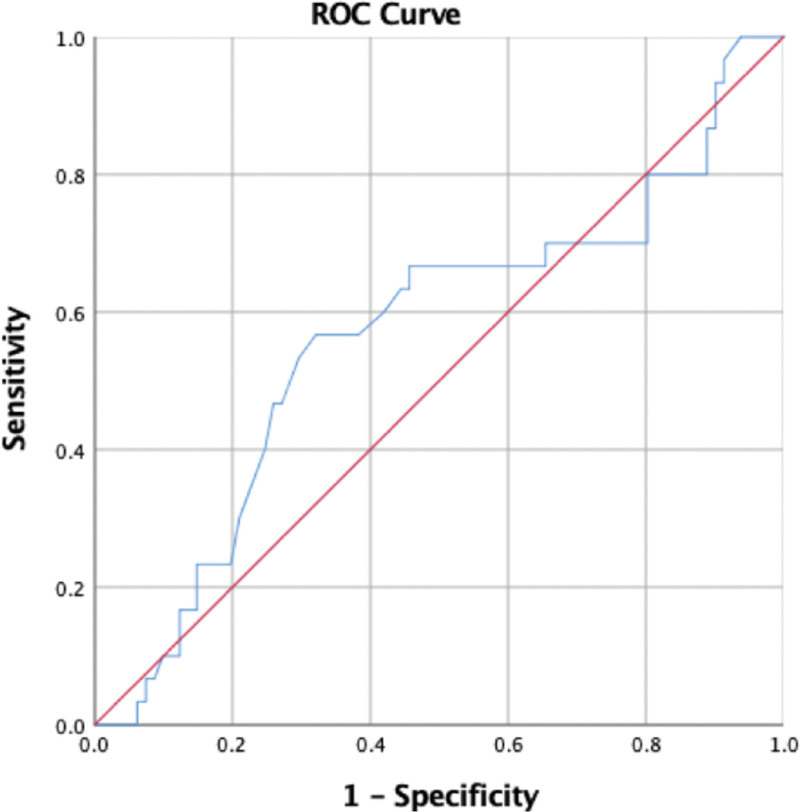
When the ROC curves of CT HU and MRI T1WI Low signal intensity are compared, the AUC is weak around 0.566, and does not show a significant *P*-value (*P* = .287). The CT-HU cutoff is low at 32.50 and has 100% sensitivity but very low specificity of 93.8%, thus rendering this ROC as one with poor predictive ability in identifying chronic rhinosinusitis. AUC = area under the curve, CT = computed tomography scan, HU = Hounsfield unit, MRI T1WI = magnetic resonance imaging T1-weighted images, ROC = receiver operator characteristic.

However, when the low-intensity signal was coded as 1 and the rest as 0, and the predictive capability of CT HU for MRI T1WI was determined, the ROC curve for T1WI value in predicting AFS showed a low predictive performance. HU yielded a low AUC value of 0.576 (95% confidence interval, 0.440 to 0.693; *P* = .287). This also resulted in higher sensitivity (68.33%) and specificity (56.67%). The Youden index was used to select the best cutoff point based on the optimal sensitivity and specificity to discriminate AFS, showing that the threshold of HU value distinguishing AFS was 32.50 HU, with a sensitivity of 100% and specificity of 93.8% (Fig. 5). Thus, rendering this ROC as one with poor predictive ability for identifying AFS.

## 4. Discussion

Before sinonasal surgery, high-resolution CT and MRI can provide valuable information regarding the surgical strategies. Opacification is frequently observed on radiographs of fungal sinus infections. Invasive fungal lesions have T2 signal voids on MRI, matching CT opacification and intrasinus or nasal fungal components.^[14]^ However, no clear criteria have been established for AFS diagnosis using CT-HU versus MRI signal intensity. In this study, we investigated the value of MRI signal intensity in evaluating AFS compared to CT Hounsfield units. Our results showed that the HU was significantly associated with MRI T2WI signal values when the signal was void only, yielding data showing that above a CT HU of 69.5, the MRI signal can be predicted with a void signal and used for the diagnostic workup and evaluation of AFS. Similar findings of low signal intensity or signal void of AFS on T2WI have been reported previously.^[14,15,19]^

In this study, the mean age was 31.9 ± 15.6 years, and most cases (66.7%) were female. Our findings revealed that AFS patients were younger, affecting more women than men, consistent with previous studies by Meng et al and Dubois et al.^[15,20]^ Individuals with AFS exhibit chronic rhinosinusitis symptoms, such as nasal congestion, sinus discomfort, and discharge. These persistent, recurring, and resistant therapies persist for over 3 months, which is consistent with our results.^[19]^

The radiologic features of noninvasive fungal sinusitis have been discussed in both radiological and otolaryngological studies. Early findings focused on bone damage, which was best assessed with CT.^[8,21]^ CT scan features are frequently considered to correspond to MRI signal intensities, resulting in the diagnosis of chronic rhinosinusitis based on subjective interpretations. High-density sinuses are frequently linked to fungal illness, although they should be assessed alongside other clinical signs if fungal disease is suspected.^[22]^ For example, hyperattenuating sinuses, nasal polyps, and serum antiseptic IgE predicted AFS with a sensitivity of 70% and a specificity of 100%. However, MRI characteristics can aid in the identification of AFS in undetermined cases.^[15,23]^

The mean HU in this study was 248.4 ± 124.4 HU (range 43.0–435.0 HU), indicating that HU values in fungal lesions were high. Generally, diseases causing opacification on images increase HU levels in affected tissues. In chronic rhinosinusitis patients, HU values in the medial wall of the maxillary sinus were lower due to osteitis, while HU values increased in individuals with greater mucosal and sinus wall thickness.^[24]^ Emre et al discovered that HU values in the medial wall of the maxillary sinus in chronic rhinosinusitis patients were 200.2 ± 24.86 HU.^[25]^ In another study, HU values were measured inside sphenoid sinus lesions, and the maximum HU in patients with chronic rhinosinusitis patients was 435.08 ± 269.33 HU.^[10]^ In another study by Zinreich et al, the reported HU for AFS was 122.2 ± 8.2 HU. The lowest representative CT number was 89.0 ± 7.84 HU; the highest was 211.4 ± 7.25 HU; and the mean was 122.2 ± 8.1 HU.^[26]^ The discrepancy in obtaining HU in different studies may be due to variations in HU values in the different sinuses and radiation exposure in CT. Generally, HU values rise in individuals with greater mucosal and sinus wall thickness.^[26–28]^

A recent review mentioned that whenever fungal material is present, the sinus content exhibits a low STIR signal and a middle-to-high signal on T1WI. The T2WI low signal or signal void is caused by metal concentration as well as high protein and low free water content.^[29]^ In our study with MRI T2WI, CT HU could be used to predict the MRI signal intensity in evaluating AFS. Receiver operating characteristic (ROC) curve analysis showed moderate predictive performance. HU yielded a significantly higher AUC value of 0.856 (95% confidence interval, 0.725 to 0.901, *P* = .001) in the ROC curve analysis. This also resulted in higher sensitivity (95.31%) and specificity (74.47%). An ideal CT HU cutoff value of 69.50 HU was obtained with a sensitivity of 100% and specificity of 44.7%. The inflamed mucosal lining of the paranasal sinuses (PNSs) also displayed hypersignal intensity thickening along the sinus walls on T2WI. However, researchers hypothesize that this thickening may be misinterpreted as nonfungal chronic sinusitis.^[14]^ Our results were similar to those of previous reports, such as Wang et al, who reported that T2WI value is a promising imaging biomarker for predicting eosinophilic chronic rhinosinusitis with nasal polyps.^[18]^ Another study examined CT and MRI results in ten individuals with AFS. All patients had significant attenuation in the central sinus on CT and T2WI MR signal voids, demonstrating that AFS is a separate clinical entity with a highly distinctive radiographic presentation.^[6]^ In a study by Wang et al, the ROC curve of T2WI value for predicting chronic rhinosinusitis had an AUC of 0.78.^[18]^ Additionally, a recent study by Meng et al reported that MRI has a high diagnostic rate (100%).^[15]^ Seo et al found that 94.1% of 17 patients had low T2WI signal intensity, while Nomura et al identified fungus balls with 100% sensitivity using hypo-signal intensity.^[30,31]^ This signal void in T2WI has been attributed to magnesium and iron, which are essential for fungal development.^[23]^

Theoretically, the T1WI value is correlated with the degree of tissue fibrosis and will differ between chronic allergic rhinosinusitis and non-chronic rhinosinusitis. However, no significant difference in T1WI signal void values was observed in our study. These results may be due to the diffuse, variable presentation of AFS in T1WI MR images,^[23]^ which varied from high to low signal intensity. Similar findings were reported by Wang et al^[18]^ and Guo et al.^[32]^ In the Guo et al study, T1WI was associated with poor predictive performance (AUC value of 0.625), suggesting the difficulty in identifying the T1WI endotype in patients with chronic rhinosinusitis with nasal polyps.^[32]^

### 4.1. Study limitations

The main limitation of this study is its reliance on secondary data, the quality of which can be inconsistent due to variations in documentation, data integrity, and record-keeping practices. Furthermore, the retrospective nature of the study and lack of a control group may have introduced inherent biases. Excluding records with incomplete data could also introduce a selection bias into the analysis. Additionally, the analysis did not compare symptom severity, suggesting a need for a more in-depth investigation to determine the predictive power of radiographic density measurement and MRI signal voids in AFS. To mitigate these limitations and provide more robust findings, we recommend conducting a prospective study with a larger sample size and a control group.

## 5. Conclusion

Our results showed that HU was significantly associated with MRI T2WI signal values when the signal was void only, yielding data showing that above a CT HU of 69.5, the MRI signal can be predicted with a void signal and used for the diagnostic workup and evaluation of fungal rhinosinusitis. Additionally, there was no correlation between HU density and MRIT1WI, and we could not rely on the T1WI signal intensity to predict fungal rhinosinusitis.

## Author contributions

**Conceptualization:** Seham Alsalem, Ali Almontashri, Mohammed Alsalem, Fahad Altamimi, Nasher Alyami, Shaker Hajjaf, Faisal Ahmed.

**Data curation:** Seham Alsalem, Ali Almontashri, Mohammed Alsalem, Fahad Altamimi, Nasher Alyami, Faisal Ahmed.

**Formal analysis:** Seham Alsalem, Ali Almontashri, Mohammed Alsalem, Fahad Altamimi, Shaker Hajjaf, Faisal Ahmed.

**Funding acquisition:** Seham Alsalem, Ali Almontashri, Mohammed Alsalem, Nasher Alyami, Shaker Hajjaf.

**Investigation:** Seham Alsalem, Ali Almontashri, Mohammed Alsalem, Fahad Altamimi, Nasher Alyami, Faisal Ahmed.

**Methodology:** Seham Alsalem, Ali Almontashri, Mohammed Alsalem, Fahad Altamimi, Nasher Alyami, Shaker Hajjaf, Faisal Ahmed.

**Project administration:** Seham Alsalem, Ali Almontashri, Shaker Hajjaf.

**Resources:** Seham Alsalem, Fahad Altamimi, Nasher Alyami.

**Software:** Seham Alsalem, Ali Almontashri, Mohammed Alsalem, Fahad Altamimi, Nasher Alyami, Faisal Ahmed.

**Supervision:** Seham Alsalem, Ali Almontashri, Mohammed Alsalem, Fahad Altamimi, Faisal Ahmed.

**Validation:** Seham Alsalem, Fahad Altamimi, Faisal Ahmed.

**Visualization:** Seham Alsalem, Fahad Altamimi, Shaker Hajjaf.

**Writing – original draft:** Seham Alsalem, Ali Almontashri, Mohammed Alsalem, Fahad Altamimi, Nasher Alyami, Faisal Ahmed.

**Writing – review & editing:** Seham Alsalem, Fahad Altamimi, Faisal Ahmed.

## References

[1] Schubert MS. Allergic fungal sinusitis: pathophysiology, diagnosis and management. Med Mycol. 2009;47(Suppl 1):S324–30. 19330659 10.1080/13693780802314809

[2] Ni Mhurchu E Ospina J Janjua AS Shewchuk JR Vertinsky AT. Fungal rhinosinusitis: a radiological review with intraoperative correlation. Can Assoc Radiol J. 2017;68:178–86. 28438285 10.1016/j.carj.2016.12.009

[3] Chakrabarti A Denning DW Ferguson BJ. Fungal rhinosinusitis: a categorization and definitional schema addressing current controversies. Laryngoscope. 2009;119:1809–18. 19544383 10.1002/lary.20520 PMC2741302

[4] Al-Qahtani K Altamimi FN Al-Harbi MH Islam T Al-Zendi NA Aldajani NF. The evaluation of the sensitivity and specificity of a new endoscopic diagnostic sign of allergic fungal rhinosinusitis: intrapolypoidal white particles. J Maxillofac Oral Surg. 2021;20:612–8. 34776694 10.1007/s12663-020-01357-4 PMC8554882

[5] John DS Shyam K Andrew D Cicilet S Deepalam SR. Utilizing CT soft-tissue markers as a screening tool for acute invasive fungal sinusitis. Br J Radiol. 2022;95:20210749. 34919410 10.1259/bjr.20210749 PMC9153695

[6] Manning SC Merkel M Kriesel K Vuitch F Marple B. Computed tomography and magnetic resonance diagnosis of allergic fungal sinusitis. Laryngoscope. 1997;107:170–6. 9023239 10.1097/00005537-199702000-00007

[7] Salamah MA Alsarraj M Alsolami N Hanbazazah K Alharbi AM Khalifah W Sr. Clinical, radiological, and histopathological patterns of allergic fungal sinusitis: a single-center retrospective study. Cureus. 2020;12:e9233. 32821581 10.7759/cureus.9233 PMC7430688

[8] Al-Dousary S Alarifi I Bin Hazza’a A Sumaily I. Paranasal sinus wall erosion and expansion in allergic fungal rhinosinusitis: an image scoring system. Cureus. 2019;11:e6395. 31938671 10.7759/cureus.6395 PMC6957240

[9] Killeen DE Sedaghat AR Cunnane ME Gray ST. Objective radiographic density measurements of sinus opacities are not strong predictors of noninvasive fungal disease. Am J Rhinol Allergy. 2014;28:483–6. 25514484 10.2500/ajra.2014.28.4104

[10] Tunç O Yazici A Aytaç I Tümüklü K Akşamoğlu M. Value of Hounsfield units in the evaluation of isolated sphenoid sinus lesions. Allergy Rhinol (Providence). 2021;12:21526567211032560. 34457372 10.1177/21526567211032560 PMC8387604

[11] Huang Z Xu H Xiao N. Predictive significance of radiographic density of sinus opacity and bone thickness in unilateral maxillary sinus mycetoma. ORL J Otorhinolaryngol Relat Spec. 2019;81:111–20. 31238303 10.1159/000496829

[12] Nakayama T Miyata J Inoue N Ueki S. Allergic fungal rhinosinusitis: what we can learn from allergic bronchopulmonary mycosis. Allergol Int. 2023;72:521–9. 37442743 10.1016/j.alit.2023.06.005

[13] AlAhmari AA. Allergic fungal rhinosinusitis in Saudi Arabia: a review of recent literature. Cureus. 2021;13:e20683. 35106223 10.7759/cureus.20683 PMC8785804

[14] Kim SC Ryoo I Shin JM Suh S Jung HN Shin SU. MR findings of fungus ball: significance of high signal intensity on T1-weighted images. J Korean Med Sci. 2020;35:e22. 31950777 10.3346/jkms.2020.35.e22 PMC6970076

[15] Meng Y Zhang L Piao Y Lou H Wang K Wang C. The use of magnetic resonance imaging in differential diagnosis of allergic fungal sinusitis and eosinophilic mucin rhinosinusitis. J Thorac Dis. 2019;11:3569–77. 31559063 10.21037/jtd.2019.07.26 PMC6753439

[16] Sedaghat AR Bhattacharyya N. Chronic rhinosinusitis symptoms and computed tomography staging: improved correlation by incorporating radiographic density. Int Forum Allergy Rhinol. 2012;2:386–91. 22550029 10.1002/alr.21042

[17] Sedaghat AR Bhattacharyya N. Radiographic density profiles link frontal and anterior ethmoid sinuses behavior in chronic rhinosinusitis. Int Forum Allergy Rhinol. 2012;2:496–500. 22736637 10.1002/alr.21063

[18] Wang Y Lou H Xian M. Investigation of the value of T 2 mapping in the prediction of eosinophilic chronic rhinosinusitis with nasal polyps. J Comput Assist Tomogr. 2023;47:329–36. 36723408 10.1097/RCT.0000000000001411 PMC10045955

[19] Masmoudi M Chelli J Ben Mabrouk A. Noninvasive fungal rhinosinusitis: a case series. F1000Res. 2021;10:869. 36225239 10.12688/f1000research.67204.1 PMC9525990

[20] Dubois A Simon F Alanio A. Allergic fungal rhinosinusitis and eosinophilic mucin chronic rhinosinusitis: differential diagnostic criteria. A two-center comparative study following STROBE methodology. Eur Ann Otorhinolaryngol Head Neck Dis. 2023;140:267–70. 37833161 10.1016/j.anorl.2023.10.007

[21] Sanghvi D Kale H. Imaging of COVID-19-associated craniofacial mucormycosis: a black and white review of the “black fungus.”. Clin Radiol. 2021;76:812–9. 34364672 10.1016/j.crad.2021.07.004 PMC8316064

[22] Ilica AT Mossa-Basha M Maluf F Izbudak I Aygun N. Clinical and radiologic features of fungal diseases of the paranasal sinuses. J Comput Assist Tomogr. 2012;36:570–6. 22992608 10.1097/RCT.0b013e318263148c

[23] Mossa-Basha M Ilica AT Maluf F Karakoç O Izbudak I Aygün N. The many faces of fungal disease of the paranasal sinuses: CT and MRI findings. Diagn Interv Radiol. 2013;19:195–200. 23271503 10.5152/dir.2012.003

[24] Sonone J Nagpure PS Puttewar M Garg D. Changes in maxillary sinus volume and it’s walls thickness due to chronic rhinosinusitis: a prospective study. Indian J Otolaryngol Head Neck Surg. 2019;71(Suppl 3):2182–5. 31763317 10.1007/s12070-019-01613-1 PMC6848555

[25] Emre IE Celebi I Ercan I. The radiologic evaluation of osteitis type and formation in chronic rhinosinusitis with and without nasal polyposis. Am J Rhinol Allergy. 2015;29:e201–4. 26637570 10.2500/ajra.2015.29.4240

[26] Zinreich SJ Kennedy DW Malat J. Fungal sinusitis: diagnosis with CT and MR imaging. Radiology. 1988;169:439–44. 3174990 10.1148/radiology.169.2.3174990

[27] Moss WJ Finegersh A Jafari A. Isolated sphenoid sinus opacifications: a systematic review and meta-analysis. Int Forum Allergy Rhinol. 2017;7:1201–6. 29024448 10.1002/alr.22023

[28] Lee SH Kim HJ Lee JW Yoon YH Kim YM Rha KS. Categorization and clinicopathological features of chronic rhinosinusitis with eosinophilic mucin in a korean population. Clin Exp Otorhinolaryngol. 2015;8:39–45. 25729494 10.3342/ceo.2015.8.1.39 PMC4338090

[29] Tostes VS de Oliveira Schiavon JL Lederman HM. Fungal sinusitis: magnetic resonance image findings on immunocompromised patients. Curr Radiol Rep. 2017;5:9.

[30] Seo YJ Kim J Kim K Lee JG Kim CH Yoon JH. Radiologic characteristics of sinonasal fungus ball: an analysis of 119 cases. Acta Radiol. 2011;52:790–5. 21525111 10.1258/ar.2011.110021

[31] Nomura K Asaka D Nakayama T. Sinus fungus ball in the Japanese population: clinical and imaging characteristics of 104 cases. Int J Otolaryngol. 2013;2013:731640. 24324499 10.1155/2013/731640 PMC3845720

[32] Guo CL Lu RY Wang CS. Identification of inflammatory endotypes by clinical characteristics and nasal secretion biomarkers in chronic rhinosinusitis with nasal polyps. Int Arch Allergy Immunol. 2023;184:955–65. 37253337 10.1159/000530193 PMC10614570

